# An acute adrenal insufficiency revealing pituitary metastases of lung cancer in an elderly patient

**DOI:** 10.11604/pamj.2016.23.34.8905

**Published:** 2016-02-08

**Authors:** Hela Marmouch, Sondes Arfa, Saoussen Cheikh Mohamed, Tensim Slim, Ines Khochtali

**Affiliations:** 1 Department of Endocrinology and Internal Medecine, Fattouma Bourguiba University Hospital, Monastir, Tunisia; 2 Department of Pneumology, Fattouma Bourguiba University Hospital, Monastir, Tunisia

**Keywords:** Pituitary metastasis, hypopituitarism, acute adrenal insufficiency, lung cancer

## Abstract

Metastases of solid tumors to the pituitary gland are often asymptomatic or appereas as with diabetes insipid us. Pituitary metastases more commonly affect the posterior lobe and the infundibulum than the anterior lobe. The presentation with an acute adrenal insufficiency is a rare event. A 69-year-old men presented with vomiting, low blood pressure and hypoglycemia. Hormonal exploration confirmed a hypopituitarism. Appropriate therapy was initiated urgently. The hypothalamic-pituitary MRI showed a pituitary hypertrophy, a nodular thickening of the pituitary stalk. The chest X Rays revealed pulmonary opacity. Computed tomography scan of the chest showed a multiples tumors with mediastinal lymphadenopathy. Bronchoscopy and biopsy demonstrated a pulmonary adenocarcinoma. Hence we concluded to a lung cancer with multiple pituitary and adrenal gland metastases. This case emphasizes the need for an etiological investigation of acute adrenal insufficiency after treatment of acute phase.

## Introduction

Pituitary tumors are the most frequent intracranial neoplasm, affecting 11000 of the worldwide population [1]. However metastases in this location are rare and uncommon presentation of systemic malignancy. The clinical and radiologic features of most pituitary metastases can be characteristic and evocative but in no case pathognomic. The diabetes insipidus is the most common clinical manifestation of the disease [2, 3]. We report herein a case of an acute adrenal insufficiency revealing pituitary metastases of lung cancer.

## Patient and observation

**Figure 1:**
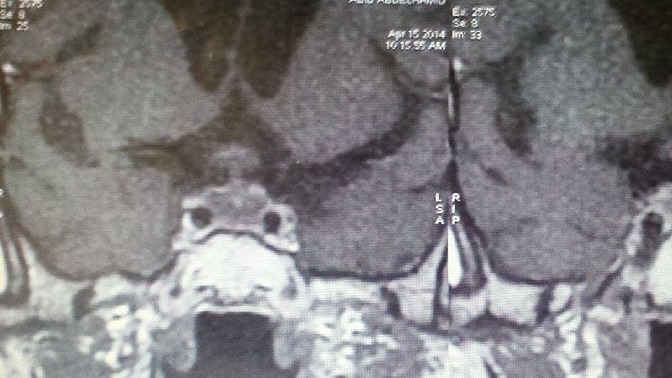
Magnetic resonance imaging: an inhomogeneous pituitary hypertrophy with convexity of the sellar diaphragm

**Figure 2:**
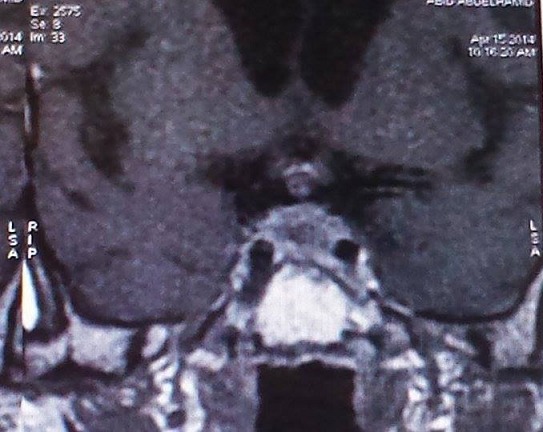
Magnetic resonance imaging: a nodular thickening of the pituitary stalk

A 69-year-old patient active smoker with history of type 2 diabetes mellitus presented with signs of acute adrenal insufficiency; vomiting, low blood pressure and hypoglycemia, associated to a polyuro polydipsic syndrom. Assessment of pituitary function revealed hypopituitarism and an insipidus diabetes; serum cortisol level of 12.6 ng/mL (normal range 100-250 ng/mL), Free T4 level of 6.8 pg/mL (normal range 8-18 pg/mL), thyroid stimulating hormone level of 0.005mUI/mL (normal range 0.5-4 IU/mL), total testosterone level of 0.025 ng/mL (normal range 2.5-10 ng/mL), follicle stimulating hormone level of 0.5 IU/mL (normal range 1-8.4 IU/mL), luteinizing hormon level of 0.1IU/L (normal range1-10.5 IU/L) and low urine osmolarity. A hormone replacement therapy was indicated urgently. In the first 12 hours after admission, a magnetic resonance imaging was performed and demonstrated an inhomogeneous pituitary hypertrophy, with convexity of the sellar diaphragm, a nodular thickening of the pituitary stalk, and a loss of high intensity signal from the posterior pituitary (Figure 1, Figure 2). The ophthalmologic examination was normal.

**Figure 3:**
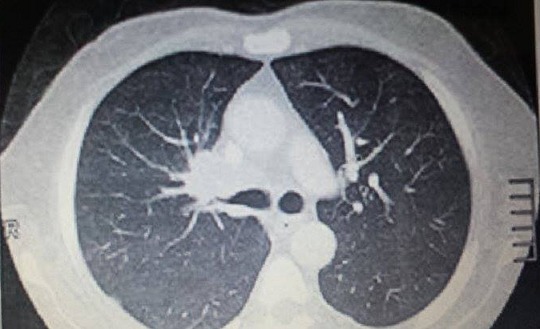
The computary tomography scans of the Chest: a mass in the upper right lobe with mediastinal lymph nodes

In front of the deterioration of the general condition, tobacco intoxication, the imagery founds and the very high level of carcinoembryonic antigen (CEA) at 197µg/L, hypothalamic and pituitary metastasis was suspected. The computary tomography scan of the Chest, Abdomen and Pelvis revealed a mass in the upper right lobe with mediastinal lymph nodes, liver and bilateral adrenal metastases (Figure 3). A biopsy with a specific treatment was planned.

**Consent:** a written informed consent was obtained from the patient for the publication of this paper and the accompanying images.

## Discussion

### Frequency

Authors of reports on large autopsy series have stated that pituitary metastases occur in between 1 and 3.6% of patients with malignant tumors, if one considers autopsy series in which both the pituitary and surrounding sella turcica have been evaluated, however, rates of metastasis as high as 27% have been reported to occur in this area [4]. Zoli et al, reported 15 cases of pituitary metastases among 1469 operated pituitary tumors (1% of total tumors). The average age at the time of surgery was 65 years (range, 46-77 years) [5]. On other hand, these autopsy series revealed that the pituitary is macroscopically normal in two thirds of cases [2]. Metastases to pituitary adenomas is a rare event; il only occurs in 1-5% patients with known malignancies. To the best of our knowledge, only 20 cases of pituitary metastases are reported in the literature. Metastases are preferentially located on the posterior pituitary. Thus, in a series of patients with pituitary metastases exclusive involvement of the posterior pituitary was noted in 52% of cases, while a breach of the anterior pituitary was present in 21% of cases (n=178). Both anterior and posterior pituitary were affected in the same time in 27% of cases [6].

### Primary tumors

Breast and lung carcinomas represent the most frequent tumors that metastasis on a normal pituitary or on a pituitary adenoma. Other primary tumors have been reported, such as prostate, renal cell, gastrointestinal cancers, lymphoma, leukemia, thyroid carcinoma, and plasmocytoma [3, 4].

### Clinical findings

The majority of pituitary metastases are clinically silent. Only 7% of pituitary metastases were symptomatic. Among symptomatic patients, diabetes insipidus is the most frequently reported finding, with a variable incidence ranging from 29% to 81%. Other symptoms are essentially represented by ophthalmoplegia, headache/pain, vomiting, visual field defects, decreased consciousness, and anterior pituitary dysfunction [7]. These symptoms can be the manifestation of a pituitary apoplexy. Although pituitary apoplexy usually occurs in patients with pre-existing pituitary macroadenoma, it has also been described in those with a normal pituitary gland, craniopharyngioma, lymphocytic hypophysitis and, in rare instances (<5%), pituitary metastasis [8]. The diagnosis is made in front of clinical signs and confirmed by with possible histopathological examination.

### Physiopathology

The explanation for metastatic tumor localization to pituitary is not well understood. In patients with metastasis to the pituitary gland, four pathways for metastatic spread to this gland have been identified, as follows: Direct blood-borne metastasis to the posterior lobe with subsequent expansion; Blood-borne metastasis to the pituitary stalk with growth into the anterior and posterior pituitary lobes; Blood-borne metastasis to the clivus, dorsum sellae, or cavernous sinus, which then spreads into the pituitary gland; Leptomeningeal spread with involvement of the pituitary capsule.

However, in patients with metastasis to a pituitary adenoma, the metastasis often takes the path of the arterial blood supply of the adenoma, the blood supply from capsular arteries originating in the internal carotid artery. Functionally, diabetes insipidus is an expression of stalk-posterior pituitary damage, and frequently it manifests at early stages, whereas anterior pituitary insufficiency may be a subsequent manifestation [5]. On an other hand, due to the inhibitory effect of glucocorticoids on the secretion of antidiuretic hormone, the substitution of steroid insufficiency caused by pituitary or a adrenal insufficiency, by Hydrocortisone can unmask a diabetes insipidus. This fact was detected in our case, where the first symptoms reveal an acute adrenal insufficiency whose treatment has uncovered a diabetes insipidus.

### Radiologic findings

MRI is the preferred technique to demonstrate pituitary metastases [8]. However, the question is how to differentiate pituitary metastasis from pituitary adenoma in patients with a history of malignant disease, but also in those in which pituitary metastasis is the initial symptom of a malignant disease. Some radiological characteristics have been reported to be helpful in differentiating pituitary metastases from pituitary adenomas; these include the following: 1) thickening of the pituitary stalk; 2) loss of a high-intensity signal from the posterior pituitary; 3) isointensity on both T1- and T2-weighted magnetic resonance images; 4) invasion of the cavernous sinus; and 5) sclerotic changes around the sella turcica. Clinically, the presence of diabetes insipidus is very suggestive of pituitary metastasis and can be the first manifestation of a malignant neoplasm. Although history or coexistence of malignancy usually leads to the diagnosis, it is of limited diagnostic value because 1.8 to 16 percent of patients with known malignancy and a sellar tumor turn out to harbor a pituitary adenoma [9].

### Treatment

Treatment, mostly palliative, depends on symptoms. It is usually limited and includes palliative radiotherapy, hormone replacement therapy when indicated and/or chemotherapy for the primary cancer. Surgical exploration and decompression, alone or combined with radiation, is often necessary when suprasellar extension causes progressive deterioration in vision and/ or pain [9].

### Prognosis

The prognosis of metastatic cancer to pituitary adenoma is grim, as most patients already have widespread metastases at the time of diagnosis. The mean survival after the development of pituitary metastasis is only six months, with an overall 1-year mortality rate of more than 90% [2]. Our patient received adjuvant chemotherapy and if possible radiotherapy on the pituitary gland.

## Conclusion

Despite the fact that pituitary metastasis are rare, they must be evoked on the presence of pituitary involvement, sudden onset of adrenal insufficiency, even in the absence of a neoplastic history. Pituitary tumor and / or metastasis should be taken in account in differential diagnosis.
